# Pancreatic Adenocarcinoma in Patients with Type 2 Diabetes: Prognosis and Survival

**DOI:** 10.7759/cureus.9382

**Published:** 2020-07-25

**Authors:** Meshari A Turjoman, Saud F Alshaikh, Alwaleed S Althobaiti, Mohammed A Yateem, Ziyad K Saifaddin, Turki M AlFayea

**Affiliations:** 1 College of Medicine, King Saud bin Abdulaziz University for Health Sciences, King Abdulaziz Medical City, National Guard Hospital, Jeddah, SAU; 2 Princess Noorah Oncology Center, King Abdulaziz Medical City, National Guard Hospital, Jeddah, SAU

**Keywords:** pancreatic adenocarcinoma, diabetes, hypertension, prognosis, survival

## Abstract

Introduction

Pancreatic adenocarcinoma and type 2 diabetes mellitus (T2DM) are inter-related. The outcomes of this association were the topic of interest of a lot of prior research in this field. The primary objective of this research is the identification of the survival rate and mortality difference between patients with pancreatic adenocarcinoma and T2DM and those without.

Methods

A retrospective observational study included 83 patients who were diagnosed and managed between 2005 and 2015 at Princess Noorah Oncology Center, King Abdulaziz Medical City, Jeddah. Patients with T2DM who were older than 18 years old and were diagnosed later with pancreatic adenocarcinoma were included.

Results

Out of 83 patients with pancreatic adenocarcinoma, 86.75% (n=72) had T2DM at the time of diagnosis. The median age at diagnosis was significantly higher than in patients without T2DM (p=0.003). The overall survival was not affected by T2DM (p=0.289). However, hypertension had a significant impact on survival rate regardless of the presence of T2DM (OR, 3.47 (95% CI: 1.09-10.98)).

Conclusion

Patients with pancreatic adenocarcinoma and T2DM were mostly women and aged around 60. T2DM did not have a significant effect on tumor profile. T2DM did not significantly affect survival, although other comorbidities, such as hypertension, did.

## Introduction

Type 2 diabetes mellitus (T2DM) is a fast-growing chronic disease with a prevalence of 8.3% worldwide [1]. In Saudi Arabia, T2DM, with a prevalence of around 24%, has been associated with a variety of malignancies, such as pancreatic adenocarcinoma, which has a prevalence of 2.5% in men and 1.5% in women [1-2]. A meta-analysis of 88 studies shows that there is a relation between pancreatic adenocarcinoma and T2DM. The relative risk of developing pancreatic adenocarcinoma was 6.69 when T2DM was diagnosed within a year. Moreover, within 10 years, the relative risk was still increased, with a relative risk of 1.36 [3]. Around 75% of pancreatic adenocarcinoma patients have T2DM at the time of diagnosis, which indicates a strong association between T2DM and pancreatic adenocarcinoma [4]. The relationship between diabetes mellitus and pancreatic adenocarcinoma remains unclear regarding the type of diabetes associated with pancreatic adenocarcinoma. Furthermore, the different pathophysiology of the two major types of diabetes mellitus has led to a variety of outcomes for each type. T2DM, found in most elderly people, is the dominant risk factor for pancreatic adenocarcinoma with a prevalence of 80%-90% in most cases [5]. As the incidence of pancreatic adenocarcinoma, along with mortality, increased, the association has obtained more and more significance [4].

T2DM has shown some effect on pancreatic adenocarcinoma treatment outcomes. Studies have shown that T2DM has a negative effect on the survival rate of patients with pancreatic adenocarcinoma after therapeutic resection [6-7]. An observational study that was done on 296 patients shows that T2DM has increased the mortality rate of pancreatic adenocarcinoma patients after resection only when T2DM was of recent onset and diagnosed before the pancreatic adenocarcinoma. However, it did not increase the mortality rate of long-standing T2DM [7]. On the other hand, the antidiabetic drug metformin, most commonly described for T2DM, is also considered an antitumor drug, as it has decreased the risk of pancreatic adenocarcinoma. In short, different pancreatic adenocarcinoma treating methods have different influences on patients with T2DM [8].

The aim of this study is to evaluate the effect of T2DM on the outcomes of pancreatic adenocarcinoma patients in terms of mortality and to assess other factors that could potentially worsen the outcomes of pancreatic adenocarcinoma in patients with T2DM.

## Materials and methods

**Figure 1:**
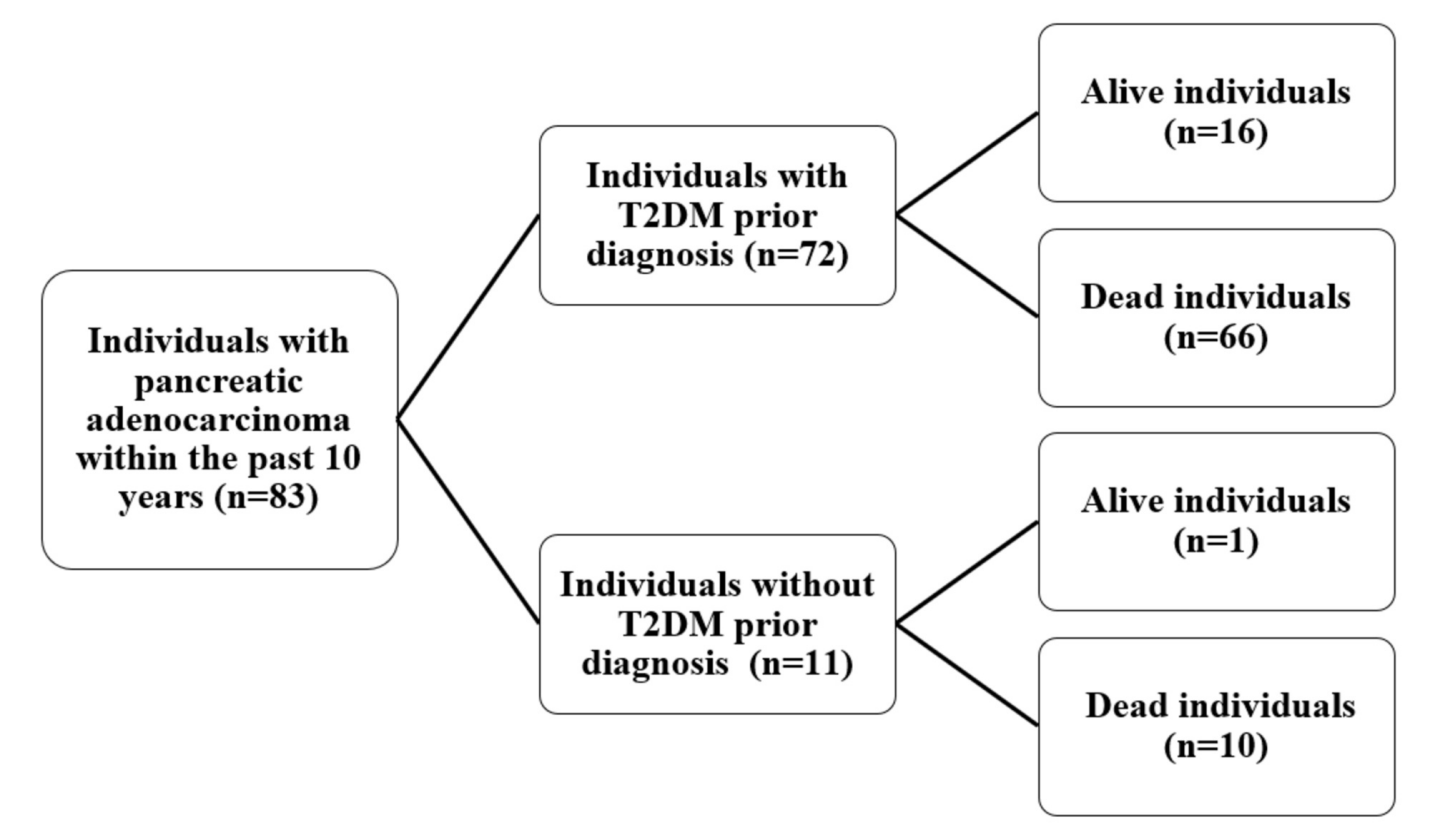
Flow chart demonstrating individuals with a prior diagnosis of T2DM and individuals without a prior diagnosis of T2DM T2DM: type 2 diabetes mellitus

We conducted a retrospective cohort study, as shown in Figure 1, at Princess Noorah Oncology Center, King Abdulaziz Medical City, Jeddah. All patients with pancreatic adenocarcinoma diagnosed in the past 10 years (2005-2015) were identified, their paper health records were obtained from the archives, and the electronic health records were taken from BESTCare, which is the health information system that was implemented in the medical records department, Ministry of National Guards - Health Affairs, King Abdulaziz Medical City, Jeddah, Saudi Arabia. Patients with T2DM, who are older than 18 years old and were diagnosed later with pancreatic adenocarcinoma, were included. We excluded patients with type I diabetes, history of prior malignancy, diagnosed with pancreatic adenocarcinoma prior to T2DM, or having been treated outside the center. Since the sample size was small, non-probability consecutive sampling was used.

The data of the patients were collected using a data collection sheet that included information about age, gender, comorbid conditions, cancer presenting symptoms, date of diagnosis (radiological and pathological), stage of cancer, type of treatment received, and the current clinical status of the patient. The comorbid conditions that were considered were: hypertension, ischemic heart disease, dyslipidemia, and chronic obstructive pulmonary disease. The cancer-presenting symptoms were: abdominal pain, dyspepsia, weight loss, nausea, and vomiting.

The ethical committee at King Abdulla Medical City had granted ethical approval for the study. As data would be collected from health records, informed consent was not applicable, and all patients’ medical record numbers were anonymized during data analysis.

We used the chi-square or Fisher exact tests for qualitative data, and the Mann-Whitney test for quantitative data. The study considered a confidence interval (CI) of 95%. The two-sided p-value of equal to or less than 0.05 was considered significant.

## Results

Cohort demographics and co-morbidities

Out of 83 patients with pancreatic adenocarcinoma, 86.75% (n=72) had T2DM at the time of diagnosis. The median age of diagnosis was 64 years old. Patients without T2DM at the time of diagnosis (n=11) had a median age of diagnosis of 49 years old (p=0.003). Forty-one patients (56.9%) who had T2DM also had co-existing hypertension. The proportion of symptoms in patients with or without T2DM was almost the same. Weight loss was higher in patients with T2DM with a percentage of 65.3% (n=47). Furthermore, nausea was also higher in patients with T2DM 48.6% (n=35). Tables 1-2 show the demographic and clinical profile of patients with and without T2DM prior to the diagnosis of pancreatic adenocarcinoma.

**Table 1:** The demographic data of patients with or without T2DM prior to the diagnosis pancreatic adenocarcinoma ǂ Using the chi-square or Fisher's exact test for qualitative data and the Mann-Whitney test for quantitative data with a 95% confidence interval T2DM: type 2 diabetes mellitus, N/A: not applicable

Parameters	Patients with pancreatic adenocarcinoma (n=83)		p-valueǂ
	T2DM (n=72)	No T2DM (n=11)	
Median Age (Range)	64 (44-94)	49 (37-78)	0.003
Gender (%)			
Male	26 (36.1%)	5 (45.5%)	.390
Female	46 (63.9%)	6 (54.5%)	
Nationality (%)			
Saudi Nationals	71 (98.6%)	11 (100%)	N/A
Non-Saudi Nationals	1 (1.4%)	0 (0%)	

**Table 2:** The clinical profile of patients with or without T2DM prior to the diagnosis of pancreatic adenocarcinoma ǂ Using the chi-square or Fisher's exact test for qualitative data and the Mann-Whitney test for quantitative data with a 95% confidence interval T2DM: type 2 diabetes mellitus, N/A: not applicable

Parameters	Patients with pancreatic adenocarcinoma (n=83)		p-valueǂ
	T2DM (n=72)	No T2DM (n=11)	
Co-morbid Condition			
Hypertension (%)	41 (56.9%)	3 (27.3%)	.065
Ischemic Heart Disease (%)	14 (19.4%)	0 (0%)	N/A
Dyslipidemia (%)	7 (9.7%)	0 (0%)	N/A
Cerebrovascular Accident (%)	2 (2.8%)	0 (0%)	N/A
Co-Malignancy (%)	2 (2.8%)	1 (9.1%)	N/A
Presenting Symptoms			
Abdominal Pain (%)	59 (81.9%)	7 (63.6%)	.157
Weight Loss (%)	47 (65.3%)	4 (36.4%)	.068
Nausea (%)	35 (48.6%)	2 (18.2%)	.056
Vomiting (%)	32 (44.4%)	3 (27.3%)	.230
Obstructive Jaundice (%)	26 (36.1%)	4 (36.4%)	.617
Other Symptoms (%)	9 (12.5%)	2 (18.2%)	.447

Outcome and survival 

Table 3 represents the tumor profile and outcome of patients with pancreatic adenocarcinoma. There was no significant statistical relationship on the staging of pancreatic adenocarcinoma between patients with or without T2DM based on tumor profile. Most of the patients with pancreatic adenocarcinoma were at a high stage at the time of diagnosis. Therefore, palliative care was the mainstay of treatment in both groups (81.8% vs. 51.4%). Palliative care included surgery such as endoscopic retrograde cholangiopancreatography (ERCP) with the intent of relieving symptoms.

**Table 3:** Tumor profile and outcome of patients with or without T2DM prior to the diagnosis of pancreatic adenocarcinoma ǂ Using the chi-square or Fisher's exact test with a 95% confidence interval T2DM: type 2 diabetes mellitus, N/A: not applicable

Parameters	Patients with pancreatic adenocarcinoma (n=83)		p-valueǂ
	T2DM (n=72)	No T2DM (n=11)	
Survival Status (%)			
Dead/Loss of Follow-Up	56 (77.8%)	10 (90.9%)	.289
Alive	16 (22.2%)	1 (9.1%)	
Cancer Stage (%)			
Resectable	8 (11.1%)	0 (0.0%)	.478
Locally Advanced	20 (27.8%)	4 (36.4%)	
Metastasis	44 (61.1%)	7 (63.6%)	
Initial Treatment (%)			
Surgery	10 (13.9%)	0	N/A
Chemotherapy	25 (34.7%)	2 (18.2%)	
Palliative Care	37 (51.4%)	9 (81.8%)	

Table 4 has shown that co-existing hypertension has a significant reduction in survival rate (odds ratio, 3.47 (95% CI: 1.09-10.98)). Out of the people who died (n=66), patients with hypertension were about 59.1% (n=39). The overall survival was not affected by T2DM (p=0.289). Dead patients (n=66) had commonly presented with abdominal pain (83.3%, n=55).

**Table 4:** Descriptive statistics, odds ratio (OR), and corresponding 95% confidence interval (CI) comparing patients with pancreatic adenocarcinoma who survived and who died ǂ Using the student's t-test for quantitative variables and the chi-square or Fisher's exact test for qualitative variables with 95% CI SD: standard deviation, N/A = not applicable

Parameters	Patients with Pancreatic Adenocarcinoma n=83		p-valueǂ	Binary Logistic Regression Analysis			
	Died n= 66	Survived n= 17		Crude OR	Significance		CI
Demographic Data							
Mean Age in Years (SD)	63 (±12)	61.9 (±10)	0.666				
Gender (%)							
Men	23 (34.8%)	8 (47.1%)	.257				
Women	43 (65.2%)	9 (52.9%)					
Co-morbid Condition							
Hypertension (%)	39 (59.1%)	5 (29.4%)	.027	3.47		0.035	1.09-10.98
Ischemic Heart Disease (%)	13 (19.7%)	1 (5.9%)	N/A				
Dyslipidemia (%)	5 (7.6%)	2 (11.8%)	N/A				
Presenting Symptoms							
Abdominal Pain (%)	55 (83.3%)	11 (64.7%)	.091				
Weight Loss (%)	39 (59.1%)	12 (70.6%)	.281				
Nausea (%)	27 (40.9%)	10 (58.8%)	.147				
Obstructive Jaundice (%)	25 (37.9%)	5 (29.4%)	.363				
Tumor Profile							
Tumor Stage (%)							
Resectable	7 (10.6%)	2 (11.8%)	N/A				
Locally Advanced	17 (25.8%)	8 (47.1%)					
Chemotherapy	42 (63.6%)	7 (41.2%)					
Initial Treatment (%)							
Surgery	7 (10.6%)	3 (17.7%)	N/A				
Chemotherapy	22 (33.3%)	5 (29.4%)					
Palliative Care	37 (56.1%)	9 (53.1%)					

## Discussion

The effect of T2DM in patients with pancreatic adenocarcinoma has been under investigation. The risk of postoperative complications for patients with T2DM and pancreatic adenocarcinoma who underwent resection was high in the long term [6]. The short-term outcome was also affected by lower survival [9]. However, our study did not show any survival advantage for patients with or without T2DM (p=0.289). To some extent, Hwang et al. have shown similar results in which pancreatic adenocarcinoma survival is not affected by T2DM in the short term (p=0.620). Nevertheless, their secondary analysis showed that in patients with T2DM, for a duration longer than five years, had a significantly worse mortality rate (p < 0.05) [10]. The inconsistency in these results has been attributed to the difference in the demographic characteristics and co-founders between the studies [7].

Other factors can have an impact on the survival rate. One study has emphasized the effect of smoking on survival, which was significant when comparing current smokers with never smokers (P =0.003) [11]. Zhang et al.'s study explored the effect of hypertension on survival. They found that there was a reduction in short-term survival (p=0.031) [9]. Furthermore, our data heavily supports this, as almost 60% of patients who died had hypertension (p=0.027).

We found out that the incidence of pancreatic adenocarcinoma in women was higher than in men. This has only been true in cases with T2DM, however, patients without T2DM had an almost equal incidence based on gender. In contrast, as reported by the Saudi Health Council, men had a higher incidence than women in 2010 [2]. A foreign study with a series of 308 cases found that the incidence difference between genders was equal [12].

The most common clinical features at the initial presentation of pancreatic adenocarcinoma include obstructive jaundice, abdominal pain, and weight loss, however, asymptomatic presentation is not uncommon [13]. In our study, on the contrary, patients with T2DM had more weight loss, abdominal pain, and nausea, with percentages of 65.3%, 48.6%, and 81%, respectively. This may be attributed to the late presentation with advancing pancreatic adenocarcinoma. Other presentations were comparable to patients without T2DM. 

Our study has limitations to be considered. First, it was a small size, single-center study. Second, the lack of data in medical records created an obstacle for data collection, especially diabetes management. Third, we did not use the Charlson Comorbidity Index to elaborate more in the comorbidities section during data collection (yes vs. no). Finally, our study mainly consisted of Saudi nationals. Despite these limitations, our study has also a few strong points. We collected data from 2005-2015 to increase the sample size. We also included comorbidities into consideration to find an association. Multicenter research, including patients from different regions, with a large sample and a comprehensive collection of data regarding all aspects of smoking, T2DM, hypertension, and other impactful factors, is needed to provide a clinical recommendation to improve survivability for patients with pancreatic adenocarcinoma.

## Conclusions

Patients with pancreatic adenocarcinoma and T2DM were mostly women and aged around 60. They mostly presented with nausea, weight loss, and abdominal pain. T2DM did not have a significant effect on tumor profile. T2DM also did not significantly affect survival, although other comorbidities, such as hypertension, did. We recommend further research into this topic to possibly raise awareness and to assess whether tighter management over comorbidities and other factors that are associated with worse outcomes will improve survival.
